# The cloning and CRISPR/Cas9‐mediated mutagenesis of a male sterility gene *MS1* of soybean

**DOI:** 10.1111/pbi.13601

**Published:** 2021-05-04

**Authors:** Bingjun Jiang, Li Chen, Chunyan Yang, Tingting Wu, Shan Yuan, Cunxiang Wu, Mengchen Zhang, Junyi Gai, Tianfu Han, Wensheng Hou, Shi Sun

**Affiliations:** ^1^ Ministry of Agriculture and Rural Affairs Key Lab of Soybean Biology (Beijing) Institute of Crop Sciences Chinese Academy of Agricultural Sciences Beijing China; ^2^ Key Laboratory of Crop Genetics and Breeding of Hebei Institute of Cereal and Oil Crops Hebei Academy of Agriculture and Forestry Sciences Shijiazhuang China; ^3^ National Center for Soybean Improvement Nanjing Agricultural University Nanjing China

**Keywords:** soybean, *GmMS1*, NACK2, CRISPR/Cas9, *male sterility 1*

Soybean is a typical photoperiod‐sensitive self‐pollinated crop. Many efforts have been made to increase the overlap in flowering periods to support introgression and integration of agronomically useful genetic materials from different ecotypes of soybean, but the combination of low hybridization rates and a laborious manual hybridization process has kept the genetic background of soybean breeding relatively narrow.

Male sterility mutants can be used to construct out‐crossing populations and are thus valuable for both basic research and breeding applications. Of the more than 20 ms mutations reported to date in soybean (Zhao *et al*., 2019), the *ms1* mutant was the first reported recessive genic male sterile mutant for soybean (Brim and Young, 1971) and has been widely used to generate recurrent selection populations which have successfully overcome the hybridization barrier, thereby broadening soybean genetic background and facilitating the development of improved varieties (Zhao *et al*., 2006). Yang *et al*. (2014) mapped *ms1* to a region (Chr13:22,489,376‐24,602,843, containing 150 predicted genes) flanked by two SSR markers (Satt516 and Satt595). However, its molecular identity is yet unknown, which has limited deeper insights.

We launched the present study to identify the causal gene for the agriculturally impactful *ms1* sterility phenotype. Our investigative approach was based on the following idea: assuming that commercial cultivars are unlikely to have retained male sterile recessive traits, we reasoned that publicly available resequencing data actually represent a ‘public control bulk’ that can be used to rapidly identify the casual loci for recessive male sterility traits in a recurrent population. In other words, our approach was distinct from conventional bulked segregant analysis and from conventional bi‐parent mapping strategies: we leveraged public resequencing data sets for soybean commercial cultivars (NCBI: SRP062560, SRP045129) (Zhang *et al*., 2019; Zhou *et al*., 2015) as a public control bulk and selected 195 homozygous *ms1* plants to construct the single mutant bulk. This bulk was whole‐genome‐resequenced using the Illumina HiSeq platform (data deposited in the NCBI SRA database with the accession number PRJNA682495). After removing duplicated reads, the effective sequencing depth was >150×. Moreover, the whole genome was virtually seamlessly covered by clean reads (Figure 1a), and 4,540,324 polymorphism sites were called.

**Figure 1:**
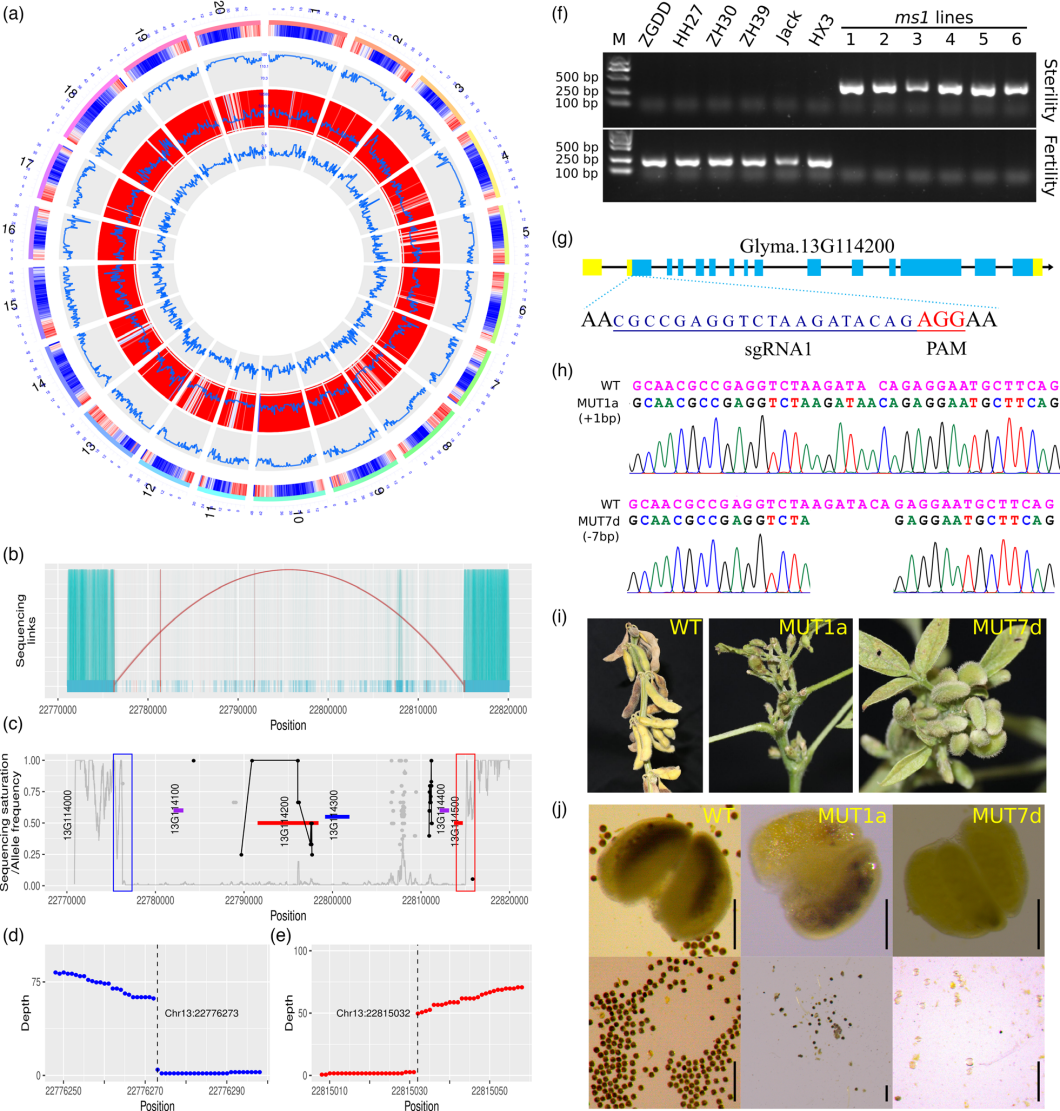
*Glyma.13G114200* is identified as the causal gene *GmMS1* for the *male sterility 1* (*ms1*) locus in soybean. (a) The entire genome of a *ms1* recurrent selection population was sequenced with high quality. From the outside in, the circles show the gene density of soybean reference genome, the average sequencing depth, and the density and the average alternative allele frequency of polymorphic sites in a 50‐kbp window, respectively. (b–e) A large fragment deletion (Chr13:22776273‐22815032) was found to be responsible for the *ms1* mutant of soybean. (b) Read mapping around the *ms1* fragment deletion. Properly paired and unproperly paired reads are linked with green and red lines, respectively. (c) Sequencing saturation and alternative allele frequency around the *ms1* fragment deletion. Grey line, sequencing saturation; coloured lines show gene locations; black ovals show alternative alleles in the gene body and the surrounding 5 kbp regions; and grey ovals show alternative alleles outside of the gene body and the surrounding 5 kbp regions. Oval height corresponds to alternative allele frequency; rectangles indicate breakpoint regions at Chr13:22776273 and Chr13:22815032 which were further detailed in (d) and (e), respectively. (f) The *ms1* fragment deletion only exists in male sterile materials but not in cultivars through regular PCR analysis. The PCR products were amplified with primer pairs FERTILITY‐F (GACGACCTTGTTGAGTCGAGA) and FERTILITY‐R (ATGAAGTTTGATGGTTCACGTACTA) and STERILITY‐F (CACCATCACCACTAGTATCACTTTTATTAC) and STERILITY‐R (AAGTTGTGTGATTGCCCAGCAAC). M, Trans2K DNA Marker; ZGDD, HH27, ZH30, ZH39, Jack and HX3, soybean cultivars Zigongdongdou, Heihe 27, Zhonghuang 30, Zhonghuang 39, Jack and Huaxia 3. (g‐j) Knockout of *Glyma.13G114200* phenocopied *ms1* materials. (g) sgRNA designed for CRISPR/Cas9 editing of *Glyma.13G114200*. (h) Knockout of *Glyma.13G114200* was verified by gold‐standard Sanger sequencing in two independent lines, MUT1a and MUT7d. (i) *Glyma.13G114200* knockout resulted in the male sterility trait. (j) Pollen from male sterile MUT1a and MUT7d plants. WT, soybean cultivar Jack. Bar, 200 μM.

Considering the mutant bulk comprising *ms1* homozygous lines, polymorphisms should be newly found with a minor allele frequency (MAF) as zero for the *ms1* casual gene. However, after detailed consideration of no polymorphic loci meeting these requirements, it became clear that small variations such as SNPs or indels were apparently not the cause of male sterility in the *ms1* mutant.

We therefore examined potential structure variations including present–absent variations (PAVs), and to effectively eliminate any enlarging effect from over‐represented regions (*e.g*. low‐complexity repeats), we set an average depth of 150× as an upper threshold with a sequencing saturation level as 1.0. This analysis identified five genes (*Glyma.13G114100*, *Glyma.13G114200*, *Glyma.13G114300*, *Glyma.13G114400* and *Glyma.13G114500*) were hardly covered by clean reads (sequencing saturation < 0.03), which are located in the previously proposed region for the *ms1* locus (Yang *et al*., 2014); in other words, these genes were absent in the *ms1* mutant bulk. Moreover, we detected a large fragment deletion (Chr13:22,776,273‐22,815,032) in the vicinity of these genes, which we further confirmed based on analysis of breakpoints (Figure 1b‐e). Consistently, DNA‐based presence/absence agarose gel electrophoresis band analysis showed that the large fragment deletion only existed in male sterile materials but not in cultivars (Figure 1f).

Of these five genes, *Glyma.13G114200* has no dysfunctional polymorphisms found in the public control bulk. Moreover, it putatively encodes a microtubule motor kinesin‐7 family protein with 950 amino acids and is a homologue of AtNACK2 which is known to be essential for phragmoplast expansion prior to cytokinesis in Arabidopsis. Moreover, AtNACK2 is expressed in the male sporophytic tissue and its mutants also exhibit male sterility (Naito and Goshima, 2015; Tanaka *et al*., 2004). Given the known cytokinesis failure after telophase II of meiosis in *ms1* mutant plants (Albertsen and Palmer, 1979), *Glyma.13G114200* was selected as the most likely causal gene for the *ms1* locus.

Using the CRISPR/Cas9 gene‐editing technology, we successfully generated two homozygous editing events: one with a single‐nucleotide insertion (MUT1a) and the other with seven bases deleted (MUT7d) (Figure 1g‐j). Both predictively resulted in two short premature proteins with 61 and 38 amino acids, respectively. Consistent with the idea of *Glyma.13G114200* locus as the casual gene for *ms1*, homozygous plants of both edited lines phenocopied the male sterile trait of the *ms1* mutant plants (Figure 1i). Moreover, pollen activity tests showed that whereas wild‐type plants had anthers full of viable pollen, the anthers of both edited lines were hollow and had dramatic and significant reductions in the number of viable pollen grains (Figure 1j). Together, results experimentally confirm that *Glyma.13G114200* can be understood as *GmMS1*, the casual gene of the *ms1* locus.

Although the functional mechanism of *GmMS1* still needs to be elucidated, our study is of agricultural significance. First, breeding efficiency will be significantly improved: the ability to do direct PCR for *GmMS1* makes it easy to accurately genotype *ms1* progeny lines as homozygous deletion or as heterozygotes, thereby greatly facilitating large‐scale production of hybrid progeny. That is, the ability to use homozygous deletion plants exclusively will prevent any undesired crosses from occurring with recipient individuals. We anticipate that the ability to easily perform hybridizations has the potential to break the bottleneck of narrow genetic diversity in soybean breeding. Second, academic researchers can use our MUT1a and MUT7d lines as confirmed male sterile recipients for easy crosses, or researchers can greatly facilitate their own breeding efforts by developing edited *GmMS1* knockouts in their own parental lines of interest.

Third, our discovery of *GmMS1* can greatly accelerate genetic interchange amongst different soybean cultivars. Moreover, it is even possible to envision a next‐generation hybridizing system in soybean to take full advantage of heterosis (Qi *et al*., 2020). Finally, we want to emphasize that the bulking strategy we employed—based on a public control bulk comprising public resequencing data and a single mutant bulk from a recurrent population—should be suitable for identifying causal genes of other recessive male sterility loci in recurrent populations.

In summary, in the present study we used an innovative ‘public control bulk’ alongside a single mutant bulk to identify *Glyma.13G114200* as the long‐sought causal gene *GmMS1*. Our identification of *GmMS1* will enable new opportunities for basic biological studies of soybean and perhaps other crops and seems likely to strongly promote application of male sterility to advance hybridization breeding and the pursuit of heterotic yield gains for soybean production.

## Conflict of interest

The authors declare that they have no conflicts of interest.

## Author contributions

S.S., W.H. and T.H. conceived the study. B.J. and S.S. designed the analyses. B.J. performed the data analysis. L.C. and S.Y. performed the CRISPR/Cas9 experiment. M.Z. and J.G. provided the *ms1* materials. S.S., C.Y. and T.W. maintained the *ms1* recurrent population. B.J. drafted the manuscripts. All authors contributed to writing the final manuscript.

## References

[pbi13601-bib-0001] Albertsen, M.C. and Palmer, R.G. (1979) A comparative light‐ and electron‐microscopic study of microsporogenesis in male sterile (*ms1*) and male fertile soybeans (*Glycine max* (L.) merr.). Am. J. Bot. 66, 253–265.

[pbi13601-bib-0002] Brim, C.A. and Young, M.F. (1971) Inheritance of a male‐sterile character in soybeans. Crop Sci. 11, 564–566.

[pbi13601-bib-0003] Naito, H. and Goshima, G. (2015) NACK kinesin is required for metaphase chromosome alignment and cytokinesis in the moss *Physcomitrella patens* . Cell Struct Funct. 40, 31–41. 25748359 10.1247/csf.14016

[pbi13601-bib-0004] Qi, X. , Zhang, C. , Zhu, J. , Liu, C. , Huang, C. , Li, X. and Xie, C. (2020) Genome editing enables next‐generation hybrid seed production technology. Mol. Plant 13, 1262–1269. 32645290 10.1016/j.molp.2020.06.003

[pbi13601-bib-0005] Tanaka, H. , Ishikawa, M. , Kitamura, S. , Takahashi, Y. , Soyano, T. , Machida, C. and Machida, Y. (2004) The *AtNACK1*/*HINKEL* and *STUD*/*TETRASPORE*/*AtNACK2* genes, which encode functionally redundant kinesins, are essential for cytokinesis in Arabidopsis. Genes Cells 9, 1199–1211. 15569152 10.1111/j.1365-2443.2004.00798.x

[pbi13601-bib-0006] Yang, Y. , Speth, B.D. , Boonyoo, N. , Baumert, E. , Atkinson, T.R. , Palmer, R.G. and Sandhu, D. (2014) Molecular mapping of three male‐sterile, female‐fertile mutants and generation of a comprehensive map of all known male sterility genes in soybean. Genome 57, 155–160. 24814801 10.1139/gen-2014-0018

[pbi13601-bib-0007] Zhang, T. , Wu, T. , Wang, L. , Jiang, B. , Zhen, C. , Yuan, S. , Hou, W. *et al*.(2019) A combined linkage and GWAS analysis identifies QTLs linked to soybean seed protein and oil content. Int. J. Mol. Sci. 20, 5915. 31775326 10.3390/ijms20235915 PMC6928826

[pbi13601-bib-0008] Zhao, Q. , Tong, Y. , Yang, C. , Yang, Y. and Zhang, M. (2019) Identification and mapping of a new soybean male‐sterile gene, *mst‐M* . Front Plant Sci. 10, 94. 30787940 10.3389/fpls.2019.00094 PMC6372514

[pbi13601-bib-0009] Zhao, S. , Zhang, M. , Jiang, C. , Yang, C. , Liu, B. and Cui, J. (2006) Study on quality improvement effect and separate character of soybean male sterile (ms*1*) recurrent selection population. Sci. Agric. Sin. 39, 2422–2427.

[pbi13601-bib-0010] Zhou, Z. , Jiang, Y. , Wang, Z. , Gou, Z. , Lyu, J. , Li, W. , Yu, Y. *et al*.(2015) Resequencing 302 wild and cultivated accessions identifies genes related to domestication and improvement in soybean. Nat. Biotechnol. 33, 408–414. 25643055 10.1038/nbt.3096

